# How gender norms affect anemia in select villages in rural Odisha, India: A qualitative study

**DOI:** 10.1016/j.nut.2021.111159

**Published:** 2021-06

**Authors:** Erica Sedlander, Sameera Talegawkar, Rohini Ganjoo, Chandni Ladwa, Loretta DiPietro, Aika Aluc, Rajiv N. Rimal

**Affiliations:** a Milken Institute School of Public Health, Department of Prevention and Community Health, The George Washington University, Washington DC, USA; b Milken Institute School of Public Health, Department of Exercise and Nutrition Sciences, The George Washington University, Washington DC, USA; c School of Medicine and Health Sciences, Department of Biomedical Laboratory Sciences, The George Washington University, Washington DC, USA; d Department of Health, Behavior and Society, Johns Hopkins Bloomberg School of Public Health, Baltimore, Maryland, USA

**Keywords:** Gender norms, Anemia, India, Qualitative

## Abstract

**Background:**

In India, 50% of women of reproductive age, compared with 23% of men, have iron deficiency anemia. Extant research focuses on biological, not social, determinants of this disparity.

**Objectives:**

The aim of this study was to examine how gender norms may affect anemia prevalence among women in rural India.

**Methods:**

We conducted 16 focus group discussions (N = 124) with women of reproductive age, husbands, and mothers-in-law and 25 key informant interviews in four villages in Odisha, India.

**Results:**

We identified the following themes that help explain how inequitable gender norms exacerbate anemia among women from different castes and tribes:

Due to a double burden of work outside the home and completing the majority of unpaid work in the home, women lack time to visit health centers to get tested for anemia and to obtain iron supplements.

Women are expected to prioritize the health of their family over their own, thus affecting their access to health care.

Women's autonomy to leave the house to seek health care is limited.

Men are the primary breadwinners for the family, but often spend their money on alcohol, rather than on iron-rich food for the household.

Intra-household food allocation favors men, in-laws, and children, thus women serve their family first, often being left with little food.

**Conclusion:**

Anemia reduction interventions need to include examination of the whole social context to successfully increase iron supplement use and iron-rich food intake. Understanding how gender norms contribute to anemia could change the narrative from a biomedical to a social justice issue.

## Highlights

• In India, more than half of women have anemia compared with less than one-fourth of men.
• We examined the pathways through which inequitable gender norms exacerbate this disparity.
• Addressing inequitable gender norms may be an upstream approach to reducing anemia.

## Introduction

Fifty-three percent of women and 23% of men in India between 15 and 49 y of age are anemic [1]. Although pregnant women have the highest prevalence of anemia, non-pregnant women make up the largest number of individuals with anemia. The vast majority of women in India with anemia have mild to moderate anemia, which affects both work capacity and productivity [1]. Therefore, reducing anemia may also contribute to reducing gender wage gaps and women's economic livelihood [2].

Despite several large-scale government programs to reduce anemia, prevalence has remained relatively unchanged over 15 y, with modest improvements among pregnant and lactating women [3]. These concerted efforts with little change raise the question of how to reduce the burden of anemia more effectively.

Although some of the reasons for higher rates of anemia in women compared with men are biological, including iron loss during menstruation and sharing nutrients during pregnancy, societal determinants also affect these disparities [4]. For example, although anemia is prevalent in both rural and urban areas of India, less affluent women and those with lower educational attainment are more likely to be diagnosed with anemia [1]. Anemia rates also differ by caste/tribe and women belonging to the tribal community have higher rates than their counterparts [1].

In India, gender inequalities negatively affect women through male-dominated decision making, economic and educational disparities, violent relationships, and the socialization of women to be “other oriented” at the expense of their own health [5]. This orientation can lead women to be passive in decisions regarding their own health needs to be able to focus more on others. A 2019 series from the *Lancet* journals highlighted the myriad ways that gender inequities affect women's health thus making it difficult to reach critical sustainable development goals [6]. This series calls for more research into how and why gender norms, which refer to expectations around how people of a particular gender are expected to behave, negatively affect women's health [7].

Prior research shows that inequitable gender norms may hinder the intake of iron-rich foods and iron supplements, thus contributing to a higher prevalence of anemia [8], [9], [10], [11]. However, existing research on this topic is limited, focuses on pregnant women, and to our knowledge, this is the first study to examine the specific pathways through which inequitable gender norms affect anemia among women. This study seeks to answer how and why gender norms affect the prevalence of anemia among women of reproductive age in Odisha, India. Data come from the Reduction in Anemia through Normative Innovations project, a randomized controlled trial using a social norms-based approach to reduce anemia in reproductive-age women [12,13].

## Methods

This study was approved by Institutional Review Boards at the George Washington University and the Institutional Ethics Committee at DCOR Consulting.

### Study setting

In Odisha (a coastal state in eastern India), 51% of women have anemia with a higher prevalence among women from a tribal culture and those with no schooling (NFHS, 2015–2016) [1]. The state is divided into 30 districts, one of which, Angul, is our focal district (Odisha Government, 2019). The state of Odisha has a thriving women's self-help group (SHG) program [14]. SHGs are gaining traction as critical microfinance platforms for bringing women together and strengthening their collective knowledge, economic power, and voice [15]. In Angul, pregnant women obtain iron-folic acid (IFA) from frontline health workers in the community: auxiliary nurse midwives, Anganwadi workers, and accredited social health activists (ASHAs). Both pregnant and non-pregnant women can go to their nearest health center to test for anemia, and if diagnosed with anemia, obtain free IFA at the clinic dispensary.

### Data collection modalities

We conducted 25 semistructured interviews with key informants (frontline health workers, schoolteachers, medical doctors, natural healers, and SHG leaders) because they provided a critical vantage point into the community as a whole. We also conducted 16 focus group discussions (N = 124) with men, women of reproductive age (WRA; 15–35 y of age), and mothers-in-law from the community. We stratified focus groups by village, sex, and age to reduce variation, to facilitate group discussion, and to describe a particular subgroup in depth [16].

### Instrument development

We developed the interview and focus group guides based on a review of the literature and feedback from co-investigators in India (Appendix 1). We also conducted three pilot interviews with key informants and three focus group discussions from nearby villages to ensure questions captured what we intended and were culturally relevant. We transcribed a subset of the pilot interviews and subsequently modified guides to improve clarity. Interview guides covered general questions about what women do on a typical day, their concerns and aspirations, and roles in the family and community. To explore women's social norms in a less personal and threatening way within the focus groups, we used vignettes, short stories about hypothetical characters that live in a rural village in Angul, India [17]. Vignettes can also help determine whether social sanctions exist and test emerging hypotheses about the existence of a social norm [18].

### Sampling

We used a random sampling procedure to select participants for focus group discussions. To do so, researchers first tabulated all the homes in each of the four villages and obtained the total number of eligible participants. Based on the number of participants needed for the focus groups, against a sampling frame that consisted of the entire village, we used a proportional skip pattern that began with a randomly selected initial participant to identify households from which to select every succeeding participant for each subgroup. To select key informant interviews, in most villages, there was only one health worker for each category, one natural healer, and so on. In the cases where there were more than one, we used purposive, expert sampling to select key informants [19].

### Recruitment

Trained research assistants approached focus group participants in their household, verbally explained the study, and asked if they would agree to participate. On the day of the interview, participants were asked to sign the consent form. All participants agreed to participate but a few left before completion of the focus group. Key informants were approached in their workplace (e.g., health center, school, etc.) and were subject to the same recruitment procedures.

### Data collection

Between March and May 2018, we collected formative research data from two blocks (which are administrative units below the district), Kishorenagar and Athamalik, in four villages. The sample description is shown in Table 1. Trained research assistants conducted all interviews, and we matched interviewer and interviewee on sex. Research assistants were from the state of Odisha and were fluent in Odiya. We selected four rural communities for our study to ensure a heterogeneous mix of caste and tribe. For each focus group, one member of the research staff observed the group while taking notes and researchers wrote field notes after each interview. Native speakers conducted all interviews face-to-face in Odiya; interviews were audio-recorded, our research partners transcribed the interviews in Odiya and then translated them into English for analysis. Participants also completed a demographic questionnaire.

**Table 1:** Data collection modalities

Focus groups		Key informant interviews	
Participant category	Number conducted	Participant category	Number conducted
Women of reproductive age	4	Frontline health workers (accredited social health activists, Anganwadi workers, auxiliary nurse midwives)	12
Mothers-in-law	4	Self-help group leaders	4
Adolescent girls	4	Medical doctors	4
Husbands	4	Teachers	3
		Natural healers	2
Total	16	Total	25

### Analysis

We uploaded transcripts and the codebook into NVivo v.12 for analysis. We developed a draft codebook then applied a thematic analysis to characterize and subsequently modify emergent themes through an iterative approach that combined data collection and analysis concurrently [20]. This allowed us to determine when saturation occurred, when no new themes emerged from the data. In this approach, we used specific a priori codes to identify text related to attitudes, beliefs, and norms about anemia, and we added additional codes to the codebook based on new themes that emerged during coding.

Four researchers—CL, RG, ST, and ES—were involved in the coding. To ensure consistency across coders, we held weekly online meetings and the lead author reviewed several coded transcripts from each researcher. All coders participated in regular online meetings over the course of the analysis to discuss codes, review memos (notes comprised of emerging themes and linkages), reconcile discrepancies, and compare emerging themes. The coders then met with a larger group of researchers (RR, AA, and LD) to discuss emerging themes further.

## Results

Characteristics of the sample included in the present study are shown in Table 2. We illustrate demographic characteristics by stratified groups: WRA, men, mothers-in-law, and key informants. In the present sample, 52% of WRAs completed up to high secondary school compared with 23% of men, 3% of mothers-in-law, and 38% of key informants. All participants reported that they were Hindu. More than half of WRAs belonged to the scheduled caste or tribal population and more than one-third of WRAs were part of the other backward caste. Men and mothers-in-law came from similar caste/tribes. Approximately 81% of WRAs had ever taken IFA compared with 3% of men, 23% of mothers-in-law, and 71% of key informants. Approximately 9% of WRAs were currently taking IFA compared with none of the men and mothers-in-law, and 4% of key informants.

**Table 2:** Demographic information: Focus group and key informant participants

	Women 15–35 y of age	Men	Mothers-in-law	Key informants
	(n = 64)	(n = 30)	(n = 30)	(n = 24)
	M (SD)	M (SD)	M (SD)	M (SD)
Age	22.14 (5.94)	32.23 (6.10)	54.77 (6.03)	40.63 (12.02)
	n (%)	n (%)	n (%)	n (%)
Education level*				
None	4 (6.3)	0 (0)	14 (46.7)	2 (8.3)
Up to primary	5 (7.8)	10 (33.4)	11 (36.7)	0 (0)
Up to secondary	13 (20.3)	7 (23.3)	4 (13.4)	4 (16.6)
Up to high secondary	33 (51.6)	7 (23.3)	1 (3.3)	9 (37.5)
Up to tertiary	9 (14.1)	6 (20)	0 (0)	4 (18.6)
Married	32 (50)	26 (86.7)	29 (96.7)	22 (91.7)
Religion-Hindu	64 (100)	30 (100)	30 (100)	24 (100)
Caste				
Scheduled caste/tribe	36 (56.3)	18 (60)	16 (53.4)	6 (25)
Other backward caste	22 (34.4)	10 (33.3)	13 (43.3)	14 (58.3)
Other caste	6 (9.4)	2 (6.7)	1 (3.3)	4 (16.7)
Children				
None	34 (53.1)	7 (23.3)	0 (0)	3 (12.5)
1 or 2	23 (35.9)	15 (50)	7 (23.3)	17 (70.8)
3	4 (6.3)	6 (20)	13 (43.3)	2 (8.3)
≥4	3 (4.7)	2 (6.6)	10 (33.3)	2 (8.3)
Ever taken IFA	52 (81.3)	1 (3.3)	7 (23.3)	17 (70.8)
Currently taking IFA	6 (9.4)	0 (0)	0 (0)	1 (4.2)
Diagnoses of anemia ever	15 (23.4)	1 (3.3)	5 (16.7)	9 (37.5)
Currently anemic	4 (6.3)	0 (0)	1 (3.3)	1 (4.2)

IFA, iron-folic acid; M, mean; SD, standard deviation.

⁎ Primary education includes grades 1–7; secondary education includes grades 8–10; higher secondary includes grades 11 and 12; and tertiary includes education after grade 12.

We identified five themes in the data describing how and why unequal gender norms may affect the prevalence of anemia among WRAs who belong to specific castes/tribes in rural Odisha, India.

1. Due to a double burden of work, women lack the time to visit a health center to get tested for anemia and/or obtain supplements.
2. Women are expected to prioritize the health of their family over their own, affecting their ability or desire to go to the health center.
3. Women's autonomy to leave the house to seek health care is limited.
4. Men are the primary breadwinners, but they often spend their money on alcohol, thus reducing household resources that could be spent on iron-rich food.
5. Women serve their family first and “adjust” to whatever food is left over at the end of their meals.

### Women lack time to visit a health center

Participants mentioned that women do not have sufficient time to complete their daily tasks, let alone seek medical care for anemia. One adolescent said, “They [women] spend all their time doing household work. When will they take care of themselves?” Not only are women solely responsible for household work, and “serving everyone in the family,” but many also work outside the home, contributing to subsistence farming or selling goods in the market. A medical doctor noted, for example: “They don't have time to think about their health.” This “lack of time” is indicative of the double burden that women bear to engage in both activities for generating income, in addition to the unequal roles they play in the economies of unpaid care and responsibilities within the household.

### Prioritizing family health over her own

Women face strong injunctive norms, expectations from others, to ignore their own well-being, complete their workload, and serve their family. If women ignore their own well-being to serve their family, they could ignore signs of anemia like fatigue and not make the time to visit a health center to seek care to prevent or treat anemia. One doctor stated, “They [women] don't give themselves importance. They immediately go to the hospital and bring medicines for their husbands and children and they sacrifice their own health.” We found that women's own health and well-being ranks last—after her children, husband, and in-laws. One husband said, “They will have to look after their husbands, children, and family. They forget the pain.”

Although the idea of women not prioritizing their own health was highly prevalent in our data, it was not universally so. Some women acknowledged the importance of taking care of their health. One woman said, “She has to take care of all. She has to take care of her own health along with her husband's and children's.” These outliers provide evidence that gender norms around taking care of others and ignoring self-care may be amenable to change.

### Women's autonomy to leave the house to seek health care is limited

Participants mentioned that a woman's ability to leave the house on her own differs from a man's, which affects her ability to get to the health center to get tested for anemia or to obtain iron supplements. One mother-in-law said, “Women generally don't go alone outside. When they go, they go with their husbands.” An adolescent from a focus group said, “If her husband is able to take her to the medical [center] it [getting iron supplements] will be easy.” Alternatively, she may have to rely on one of the health workers to accompany her to the medical center. As one participant said, “ASHA (health worker) can also take her … she will tell to ASHA didi (sister) and she will take her to hospital.” Addressing this gendered difference in autonomy to leave the house alone requires acknowledging a larger issue—differential household power and control.

We did hear some alternative views on this topic, particularly from individuals who believed women's lack of autonomy was changing for the better. Some participants said that women have more autonomy now to go out on their own than they did before. A natural healer said, “Another thing is a woman is not neglected here. Generally, people belonging to labor class are also very sincere nowadays in comparison to past days. If required, women can go alone to places like Boinda or Angul for marketing [about 1 h from the villages by car or bus].”

### Husbands purchasing alcohol reduces money to buy nutritious food

Given that we did not ask about substance abuse, the frequency with which men's alcohol-related behaviors came up in discussions was surprising. The amount of money that men spend on alcohol has a bearing on the amount of money left to buy iron-rich foods for the family. Participants mentioned, “Men earn money and spend it to buy alcohol and give the remaining to their wives to use for household expenses.”

Furthermore, according to a woman from a focus group, wives’ earnings were also used for alcohol. “Whatever the wife earns and he earns himself, 50% of it is spent on drinking liquor only.” Women said they buy household supplies, clothes, and food from the leftover money, “We [women] buy something if we get money. Some vegetables we get from our yards. Spinach, cucumber, and pumpkin … whatever else we sow, we eat.” Clearly, the earning and spending power lies with men.

Because men spend their incomes on alcohol and women have limited earning potential, household food purchasing may be limited to staples that are inexpensive and may be less rich in iron. A nurse midwife emphasized that iron-rich food is important in the prevention of anemia, “Where can they get food enriched with proper iron content? Only if they have nutritious food will their hemoglobin content in their blood increase. How will this be possible without nutritious food? From just taking a bit of rice only?” This finding aligns with the second point above that women are focusing on others rather than themselves, whereas husbands are often spending money in ways that do not benefit the family.

### Women often eat “last” and whatever is “left over”

Our data showed that inequitable intrahousehold food allocation influences both the quality and quantity of food that women consume. This has implications for the amount of iron-rich food that women are eating compared with other family members. One woman from a focus group mentioned, “First, I give food to my children and husband. Then, I eat. Their care is first. Then, I think about myself.”

In our data, we observed some variation in the number of families in which everyone ate at the same time, versus those in which women ate after everyone else. Some participants mentioned that men eat first because they are given more importance in the household. As one participant said, “The men in the family are served food first and after they have eaten, the women eat. In every village, men are given more importance in the family.” One woman reiterated this sentiment, “After they've finished their meals, I have my meal from the leftovers; I have to adjust.”

Other participants mentioned that eating together was something they practiced. “My husband says that one must eat when one is hungry.” “We stay as one soul. We eat together.” Key informants provided a more nuanced understanding of this behavior. A health worker stated, “Women eat the remaining food after the entire family has taken the food and those who think that everyone is equal, they eat together.”

Participants also perceived eating together as something that was more common in towns or cities. As one participant said, “What you said about eating together at the same time. … That happens in town-market areas but in village there is some system like if the husband and wife both are dining together then no food from your eatables should get shared with him or even touch his plate while eating.” *Touch complexity* also affected the ability to eat together in some of the villages. Certain families marginalize daughters-in-law by practicing touch complexity, such that if the daughter-in-law touches the food of the mother-in-law, the mother-in-law would not eat her meal indicating her extreme displeasure, thereby ensuring that the daughter-in-law ate after everyone else had. A WRA said, “If the daughter-in-law touches the food, the mother-in-law will not eat. The mother-in law will immediately leave the place without completing her meal.” Clearly, deeply entrenched cultural norms (including gender norms) around hierarchy within the family affect the eating order and quality and quantity of food consumption in WRA.

## Discussion

Our data show that due to a double burden of work, women do not have time in their schedule after completing all their responsibilities (much of it unpaid) and that they are expected to value their family's health and dietary needs above their own. Both of these findings have implications for a woman's ability or desire to seek preventive or curative treatment for anemia (e.g., taking the time to visit a health center). Furthermore, due to a general paucity of household funds, exacerbated by men spending money on alcohol, women's ability to buy iron-rich foods is greatly diminished. Finally, gender norms that women serve their family first and eat after everyone has finished doing so, further affect the amount and quality of food (potentially iron-rich foods) that women can consume.

This is not the first qualitative study to document the fact that gender norms may be affecting anemia among women in India. Chatterjee and Fernandes noted, for example, that gender norms were at the root of high anemia prevalence among pregnant women in Mumbai India [8]. Diamond-Smith also found, through a mixed-method study of pregnant, urban women in northern India, that the low status of women was associated with eating last in the family, thereby being associated with a poor diet and anemia [9]. Sedlander examined overall barriers and facilitators to taking IFA in rural Odisha and found that despite availability of IFA for pregnant women, non-pregnant women were largely ignored, and inequitable gender norms made it difficult for these women to seek preventive care and obtain IFA [11]. In 2017, the World Health Organization reported that gender norms that govern household food distribution or care practices may also contribute to gender differences in anemia [21]. Similar to Gupta et al., our findings show that among rural women, daughters-in-law hold the lowest-ranking position in the household and the intersection of younger age and sex put women in a particularly vulnerable position [22].

Several studies also demonstrated that women's empowerment (conceptualized as choices, control, and power for women within the household or society) was significantly associated with nutritional status. Similar to our study, Sethuraman et al. included women from the scheduled caste, scheduled tribe, and other backward caste [23]. They found that in Karnataka, southern rural India, women's socioeconomic factors did not account for their maternal nutritional well-being but that their empowerment status did with women from the tribal community reporting more empowerment than their non-tribal counterparts. Sethuraman et al. call for more studies on the nexus between women's empowerment and nutrition outcomes [23]. Similarly, a study in 48 low- and middle-income countries examined associations between gender inequities and anemia among children <5 y of age [24]. After adjusting for a country's wealth level and maternal biological and demographic factors, gender inequity showed a significant independent and positive association with anemia prevalence, explaining 50% of the variance between countries. Haas and Brownlie evaluated the evidence between iron deficiency and reduced work capacity and found strong evidence to indicate that iron deficiency anemia affected work capacity, which had downstream deleterious effects on economic productivity, household, leisure time, and child care-related activities [25].

Although these studies have documented the effect of gender roles in women's poor health, the contribution of the present study to the literature is in identifying the particular pathways through which these gender-based influences occur. Gender norms-related pathways identified in this study, for example, resulted in women eating last in the home, having limited autonomy to avail themselves of medical care, and prioritizing others’ health and well-being over their own. Each of these pathways resulted in higher risks for anemia by depriving women of nutrients through their meals and reducing their treatment-seeking behaviors. These pathways, of course, are not mutually exclusive, and we recognize considerable overlap among them. However, explicitly delineating them can provide initial insights into how interventions can either work to change them or if it is not feasible (because, for example, the intervention timeline is too short), determine how best to tailor their approaches for maximum affect. For example, to address the norms around eating last in the home, interventions could model household eating behaviors in which food is equally shared with all family members, and to address lack of autonomy, interventions could promote the idea of a buddy system in which women pair up with others in their communities to travel together for medical care.

Specifically, we observed that women prioritize the health and well-being of others in their families before thinking about their own needs. This manifests not only in women eating last and not seeking medical care for themselves, but it also likely has an effect on women's sense of self-worth. Because of the deeply rooted nature of this cultural belief, it is hard to imagine interventions, particularly those on a relatively short timeline, being able to change this in a meaningful way. Nevertheless, interventions can highlight the detrimental effects that underlying behaviors can have on women in the long term and provide direct links between the health of women in the home and that of their offspring and others they care for.

We note some underlying social changes already taking place in the community. In our data, for example, a number of participants pointed to the existence of homes in which all family members ate together, thus not requiring women to eat after everyone else had done so. This suggests that it may be fruitful to understand what sets these families apart from the more traditional ones, how others perceive this practice, and what factors led to the adoption of these practices in the first place. In some ways, this is akin to a positive deviance approach, which has delineated specific factors to keep in mind in highlighting those who deviate from established norms in either positive or negative ways [26,27].

### Limitations

The present study had some limitations. First, the four rural communities we studied in Angul, Odisha may not be representative of rural India as a whole, or even urban areas of Angul. Additionally, although the caste/tribe makeup of our sample population aligned closely with the makeup of the state of Odisha, the majority of our participants were a part of the scheduled caste, scheduled tribe, and other backward caste. Therefore, we could not generalize to other rural women in India who were not represented in our sample. Future research should include additional rural areas outside of Odisha to understand whether similar trends exist across these settings. Another limitation was that we did not collect data on income because most of the women worked as daily laborers in agriculture so income is difficult to meaningfully measure. Therefore, we used education level and caste as proxies. Furthermore, we did not collect hemoglobin data, only self-reported lifetime anemia diagnosis, so we do not know how many participants were anemic at the time of the interview. However, we know from demographic health surveys, that more than half of WRAs in this region are anemic [1]. Longitudinal studies that collect data on gender norms and IFA intake are necessary to establish causality. Social desirability bias is also a possible threat to internal validity. To account for this potential bias, researchers reminded participants that the study was confidential and there were no correct answers. We also framed our questions in terms of local vignettes to minimize social desirability.

## Conclusion

In this context, gender norms play a strong role in the prevalence of anemia among women. To design interventions that align with the social context where the target population lives, it is critical to move beyond norms related to the specific behavior of interest (e.g., taking iron supplements and eating iron-rich foods) and include more upstream norms that may indirectly affect behaviors. For example, it is critical that policymakers and program planners consider the context where they are working. Our work shows that even if iron supplements are free and available, existing gender-related barriers make it difficult for women from scheduled caste, scheduled tribes, and other backward castes to access them. Additionally, frontline health workers are currently only incentivized to provide iron supplements to pregnant women in their home or at the village health and nutrition day. Incentives could expand to include all WRAs. Although this would require government buy-in and substantial resources, this systems-level approach may truly move the needle to reduce anemia among women who are not pregnant. When discussing gender-transformative interventions, it is important to note that these norms are entrenched in social systems of unequal power and gender hierarchies. Without approaches that promote critical reflection of these norms by communities, shifting norms to be more gender-equitable will be challenging.

Considering the high prevalence of anemia among Indian women, understanding how gender norms contribute to IFA use and intake of iron-rich foods could change the narrative from a biomedical to a social justice issue. The present findings show that gender inequities may be contributing to women having more than double the burden of anemia compared with men. Although menstruation and pregnancy do have biological effects, other social factors may be at play [4]. If a woman is eating less iron-rich foods than the rest of her family, if her ability to seek health care including getting tested for anemia and taking iron supplements is limited, and if her lack of autonomy over how to spend household money results in less iron-rich foods, these are all social issues potentially contributing to the burden of disease among Indian women.

## Appendix Supplementary materials

Image, application 1
