# Supplementary material for: How gender norms affect anemia in select villages in rural Odisha, India: A qualitative study
Source: Nutrition. 2021 Jun;86:None. doi: 10.1016/j.nut.2021.111159 (PMC8209141; doi:10.1016/j.nut.2021.111159)
Supplement: Supplementary file 1 [file mmc1.docx]

**Appendix 1 – Focus Group Guide**

Before Beginning the Interview:

- Welcome participant and introduce yourself

Hello, and thank you for speaking with me today. My name is ______ and I am a field researcher at DCOR. I am conducting a research study to understand the attitudes and beliefs of people in this community. Thank you so much for agreeing to participate and taking time out of your day.

Do you have any questions about the research and your participation before we start?

**Warm-up Questions**

I’m new to this community, [name District], can you tell me a little bit about it? For example, what kind of food do people eat here? And what do people do to earn money?

**Women’s Role in the Community**

What are some of the typical things that women in this community do throughout the day? (probe: things like cooking, cleaning, taking care of kids, working outside the home, spending time with her husband).

Do women in this community have enough energy or time to these things each day?

- Look after her kids?
- Work outside the home?
- Cook?
- Spend time with her husband?
- Have more intimate time with her husband?

How are men and women treated differently in this community?

For women’s life in general, are things getting better or worse?

- in her role as a mother?

- in her role as a wife?

- in her role as a daughter in law?

What are some of the concerns that women in this community have? (probe: concerns about health, money, their family, themselves, their future)

- Some people think that (woman’s name) should first take care of her husband and kids before she worries about her own health. Other people think that she should first take care of her own health.

How do you think most women in this community feel about that? What about most men?

In some families in Odisha, women eat after their husband, children and mother in law eat. In others, women eat at the same time. What do you think about that? Is this changing?

**Anemia and Iron Folic Acid (IFA) Knowledge**

(Show an IFA tablet and liquid IFA)

Can someone tell me what this is? (probe: What does it do?)

Have you ever heard of anemia?

Many people in this community may not use the word “anemia” but they may have other words or phrases to talk about anemia. Can you tell me what some of those phrases are?

Tell me a little bit about what happens when someone has anemia (use the word or phrase identified above instead of “anemia”). Tell me how this person feels or how this person acts when they have anemia.

What do you think causes anemia? (probe: Not eating enough iron rich foods?)

What do you think makes anemia go away? (Probe: Can IFA tablets help? How about changing your diet? What kind of foods might help it go away? )

**Anemia Related Behavior**

Ok, now we’re going to make up a character. She is a female aged 23 years old. What should we call her? (Ask for suggestions and decide on a name together). She just got married and is 3 months pregnant with her first child. Her doctor just told her she has anemia.

The doctor told her to take iron tablets.

- If (same woman’s name) wanted to get IFA tablets, where could she get them? How difficult is it to *get* IFA tablets?
  - Can you tell me what she likes about taking the tablets and what she doesn’t like? (PROBE by asking more likes and dislikes)
  - Apparently, some women stop taking those tablets. Can you tell me why they stop?
  - Can you tell me what good things may happen if someone takes the tablets?
  - What do you think would help her take the tablets? Do you think (woman’s name)’s husband will support her to take the tablets? Not support her to take them? Or not say anything? (Ask for examples)
  - What about (woman’s name)’s mother in law? Will she support her in taking the tablets? Why or why not?

**IFA Norms**

- Is Anemia is a problem in the community? If yes How much of a problem?
- In general, who is typically expected to take IFA tablets? (Probe: pregnant women, adolescent girls, non-pregnant women?)
- Please think about most women like (name) who live in this community. How many of them take IFA tablets? Some? Few? Most?
- Is there anyone in (woman’s name) family (or her husband’s family) that she can talk to about taking IFA tablets? How much do you think she’ll listen to what they have to say about IFA tablets?

**Information Sources**

Imagine another woman who is not pregnant but interested in learning more about her health. What should we call her? (Ask for suggestions and decide on a name together).

Where does she go for information about health related matters?

Where can she get information about pregnancy, anemia, and IFA tablets from?

- How easy is it for her to get information about pregnancy, anemia, and/or IFA tablets? What difficulties would come up when trying to get this information?
- When a woman gets IFA tablet, what kind of information does she get about them?
- Probe: Dose, when to take or why to take it?
- For those of you that participate in self-help groups, can you tell me what kind of things you discuss?

**Closing**

That is the end of the questions I have for you, but do you have anything else you’d like to add to the discussion? Any little stories about anemia or IFA use in this community?

As a reminder, please do not share anything we spoke about today with anyone outside of this group.

Any questions?

Thank you for your time.

**Highlights**

- In India, more than half of women have anemia compared to less than a quarter of men
- We examine the pathways through which inequitable gender norms exacerbate this disparity
- Addressing inequitable gender norms may be an upstream approach to reduce anemia
